# Religious schema and tolerance towards alienated groups in Indonesia

**DOI:** 10.1016/j.heliyon.2021.e07603

**Published:** 2021-07-19

**Authors:** Rahkman Ardi, David Hizkia Tobing, Gita Nuraini Agustina, Ahmad Fauzan Iswahyudi, Diah Budiarti

**Affiliations:** a Faculty of Psychology, Universitas Airlangga, Indonesia; b Department of Psychology, Universitas Udayana, Indonesia

**Keywords:** Religious schema, Tolerance, Discrimination, Alienated groups, University, Exposure

## Abstract

National discourses that are acceptable by the alienated groups determine the level of public tolerance towards those groups. This study thus examined the relationships between religious schema and tolerance of two alienated groups in Indonesia, namely, the atheists and believers in indigenous faiths. Additionally, the study explored the differences in tolerance of these two groups across university cohorts encompassing discrete social climates and curricula. This cross-sectional study involved several universities with differing demographic makeup. The analysis results revealed that the characteristics of the evaluated target group determined the significance of the associations between the dimensions of religious schema and tolerance. Moreover, students in homogeneous educational and social environments tended to exhibit low levels of tolerance towards alienated groups. This study highlighted the importance of scrutinising the functions of intergroup exposure and dialogues in improving intergroup understanding, acceptance, and tolerance within a plural society.

## 1 Introduction

A survey conducted by the Pew Research Center on ‘The Global God Divide’ evinced that Indonesia emphasised the importance of faith in God to the extent that 96% of its population stated that belief in God was necessary for the inculcation of morality and good values (Tamir et al., 2020). Islam is the major religion in the country; however, Indonesia is not a religion-based state according to its national constitution and ideology although one of the five ideological principles of the country mandates the belief in one supreme divine being (monotheism) (Mu'ti & Burhani, 2019; Ropi, 2017). Religions other than Islam may also be the majority in some provinces or cities. For example, Hinduism is the major religion in Bali; Christianity is predominant in North Sulawesi, Papua, and West Papua and Catholicism prevails in East Nusa Tenggara (Badan Pusat Statistik, 2012). Officially, the country only recognises six religions: Islam, Christianity, Catholicism, Hinduism, Buddhism, and Confucianism (Mu'ti & Burhani, 2019; Ropi, 2017).

After a long history of denying their existence (Mu'ti & Burhani, 2019; Sudarto, 2016; Syaputra and Nasution, 2020), Indonesia has recently recognised traditional faiths apart from the six state-recognised religions (Putusan Mahkamah Konstitusi, 2016). These indigenous religions are followed in several regions by minority groups whose numbers remain indeterminate. However, around 400 indigenous faiths are estimated to exist in Indonesia (United States Department of State, 2020).

Indigenous religions may finally have attained national recognition, but the opposite treatment is meted to atheists. Indonesia does not accommodate atheism because of its ideological belief in one supreme divine being (Duile, 2018; Sudarto, 2016). Silent atheists must still choose a state-recognised religion that can be registered in their residence data (Duile, 2018). Atheists do not often openly express their disbelief in the presence of divinity, but their presence is usually implicitly revealed in closed discussions or on social media pages (Farhan and van Klinken, 2020; Schäfer, 2016).

Indeed, the majority's attitudes and behaviours towards minorities often represent a nation's culture and ideology (Dijk, 2003). Dijk (2003) stated that a national ideology is a manifestation of ingroup self-schemata that reflect the values and associations of the people. Ideological exposures (e.g. religious and national ideological stimuli) tend to render individuals more submissive in following directions and conforming to the will of the ideological stimuli (Van Cappellen et al., 2011). Thus, the majority of the citizenry is likely to harbour prejudices when the national ideology and the dominant cultural discourses are inclined to neglect certain groups because the cognitive structure of the predominant cohort does not incorporate a schematic about other groups.

The doctrines adopted during the New Order and the religious dogmatism that prevailed in Indonesia increased the stereotyping of religious minorities and encouraged prejudicial attitudes against them (Duile, 2018; Schäfer, 2016). Data from the Social Progress Imperative (2018) disclosed that Indonesia scored 2.38 and was ranked 140th in terms of freedom of religion. Notably, the country also scored 7.40 and was placed 122nd in the indicator for discrimination and violence against minorities.

### 1.1 Tolerance and religious schema

The data cited above elucidate the need to investigate the issue with tolerance towards alienated groups. This study therefore attempts to fulfil this need. Tolerance is fundamental for the development of inclusive societies and democratic governments, an aim included in the global sustainable development goals (UNDP, 2020). Tolerance is defined as the willingness to extend human rights and civil liberties to all the groups deemed external (Avery, 1988) and entails justice, fairness, empathy, and the consideration of the suffering of others (Witenberg, 2007, 2019). Mummendey and Wenzel (1999) also asserted that tolerance is an open and positive mentality towards outgroups.

Cognitive schemata (Witenberg, 2019) denote significant determinants of tolerance. A schema is a cognitive structure representing knowledge about a concept, including its attributes and the relationships between its features (Fiske & Taylor, 1991, 2017). Schemata related to religion are also termed religious schemata (Streib et al., 2010) and pertain to individual representations, knowledge, and mental ascriptions about faiths and religions they practice.

A religious schema comprises three dimensions (Streib et al., 2010): the truth of texts and teachings (TTT); fairness, tolerance, and rational choice (FTR) and xenosophia/inter-religious dialogues (XENOS). TTT pertains to a religious style that literally interprets a religion based on its sacred texts. FTR relates to a religious style that emphasises openness, rational arguments and decisions, and fairness and tolerance among human beings. XENOS entails a constructive attitude towards inter-religious dialogues through which individuals can learn from each other to apprehend the ultimate ‘truth’. Such schemas underlie human cognition, beliefs, and attitudes that will help people rationalise the phenomena they encounter (Hogg and Vaughan, 2018). Thus, schematics may help people recognise outgroups in certain situations against whom they could discriminate or could choose to treat with tolerance (Witenberg, 2019).

People are likely to inherently fear unfamiliar phenomena, including outgroups (Hogg and Vaughan, 2018). Positive or negative attitudes are often determined by optimal exposure (Hogg and Vaughan, 2018; Murphy et al., 1995; Zajonc, 1968), which, in this case, includes the evaluation of whether an unfamiliar group is deemed dangerous because of the paucity of previous exposure. If the representation of an outgroup is not yet organised in an individual's cognition due to minimal contact or exposure, the individual could find it difficult to understand the outgroup and would be likely to construct a negative perception of the other. If an external minority group is perceived as a symbolic threat to an ingroup's values, beliefs, and morals, it a consensus about the dangers of that group may be reinforced within the ingroup (Hogg and Vaughan, 2018). According to Allport (1966), religion represents a focal component of the self because it is often perceived to promote security and comfort in the social environment and can also prompt people to think about the presence of threats and dangers.

This study examined the associations between the three dimensions of the religious schema (Streib et al., 2010) and tolerance towards two alienated groups in Indonesia: the believers in indigenous faiths and the atheists. The atheist group is an unorganised minority that is not tolerated by the country's ideology (Schäfer, 2016). On the contrary, the believers of indigenous faiths have recently been accorded with legal state recognition after facing a long history of discrimination in their homeland (Mu'ti & Burhani, 2019; Sudarto, 2016).

A literature search was conducted via the Scopus index on 16th April 2021, using ‘religious schema’ and ‘prejudice’, ‘dialogue’ or ‘dialogues’, ‘relation’ or ‘relations’, ‘contact’ or ‘contacts’ and ‘tolerance’ as keywords. This search yielded 27 articles. After the articles were screened, only five were found to involve Asian populations, including Nepal, India, Iran, Malaysia and Hongkong. However, Indonesia displays unique characteristics vis-à-vis the other stated countries, particularly those in which Islam is a major religion. This study's illumination of cognitive religious schemas and their relationships with tolerance in a society whose state affairs are not ruled by Islamic (Sharia) law is thus a crucial endeavour.

This study engaged students enrolled at universities that evinced specific demographic compositions and curricula. Demographic diversity (such as whether a university is religion-based and whether it is located in a big city) determines the homogeneity or heterogeneity of the opinions of members of a university. Campus life grants students opportunities to build social associations with people from different backgrounds, thus facilitating the shaping of hybrid identities (Logli, 2015). Nevertheless, the predominant discourses of some educational institutions could promote primordial, nationalist, or universal principles (Logli, 2015). The education system in Indonesia is governed by the administration of two different departments: the Ministry of Education, Culture, Research and Technology (*Kemendikbudristek*) and the Ministry of Religious Affairs (*Kemenag*). All public and private universities are managed under the purview of the *Kemendikbudristek,* while all Islamic universities are managed by the *Kemenag* (Kemenristekdikti, 2019). The different management agencies partly determine the divergences in the curricula across the types of higher education institutes.

Therefore, this study tests the following hypotheses:

H1 An association exists between religious schema and tolerance towards believers in indigenous faiths.

H2 An association exists between religious schema and tolerance towards atheists.

The current investigation also attempts to answer the following exploratory research question: Do universities with discrete social and educational environments differ in their tolerance towards alienated religious groups (i.e. indigenous faith believers and atheists)?

## 2 Materials and methods

### 2.1 Participants

Indonesia has 122 public universities, 3,171 private universities, and 1,192 religion-based universities (Kemenristekdikti, 2019). The present study engaged 761 undergraduate students enrolled in Indonesian universities. The participants were pooled from six universities across the country: 1) a public university in Bali at which most students practice Hinduism (n = 246), 2) an Islamic public university in West Nusa Tenggara (n = 133), 3) a private Islamic university in Central Java (n = 53), 4) a Catholic private university in East Java (n = 193), 5) public universities in East Java (n = 88), and 6) a private university in East Java (n = 48).

The six universities were categorised into several groups in consideration of the following three characteristics: (1) the religious atmosphere of the university, which is generally reflected by the inclusion of the religion in the institution's name and is manifested as the majority religion of its student body, 2) universities that do not mention any religion in their name and are coordinated by the *Kemendikbud* and 3) the location of the institution (urban or rural). The three aspects yielded four new classifications: 1) a university subscribing to Hinduism as its major religion (n = 246), 2) Islamic universities (n = 186), 3) a Catholic university (n = 193) and 4) universities that do not subscribe to any religion and are located in a metropolitan city in East Java (n = 136).

The study's participants were aged between 18 and 27 years (M = 19.30; SD = 1.26; 4 participants chose not to disclose their ages). The gender proportion was 76.3% female, 23% male, and the rest preferred not to divulge their gender.

Islam was the major religion and was practised by 40.3% of the participants, followed by Hinduism at 32.3%, Christianity at 14.5%, Catholicism at 11.6% and Buddhism at 1.3%. Most participants (51%) majored in social studies, law and the humanities, 27.2% were studying health sciences, 4.9% were science students, 1.4% were engineering majors and the remaining 15.5% did not reveal their disciplines. Only 44% of the participants were active in student organisations. The monthly expenditures of most participants (56.4%) were reported at IDR 0–1 million, while 39.2% spent IDR 1–3 million, 3.4% expended IDR 3–6 million and 0.8% paid more than IDR 6 million.

The initial sample planning was calculated with an *a priori* power analysis using G∗Power software (Faul et al., 2007). The calculation was applied for a linear multiple regression fixed model R^2^ deviation from zero (predictors = 3, statistical power = .80, α = .05 and *f* ^*2*^ = .02) and yielded the requirement of a minimum of 550 respondents. The small effect size was assumed because of the heterogeneity of the planned sample that would be pooled from several cities, particularly in terms of the private or public status of the universities, whether a university's education was imparted based on a certain religion and whether the students were engaged in student bodies. Eventually, the study recruited an aggregate of 761 participants. A sensitivity power analysis (α = .05, statistical power = .80, sample size = 761 and predictors = 3) indicated that the smallest detectable effect size would be *f*^*2*^ = .014.

### 2.2 Procedure

Data were collected between August and December 2019. Paper-based questionnaires were administered at a Catholic private university in Java and at an Islamic public university in West Nusa Tenggara. Online questionnaires were employed for the four remaining universities. Purposive access was obtained to the six universities through colleagues of the researchers at these universities. Clearance was obtained for several undergraduate classes for online or paper-based data collection. Every questionnaire was completed in the presence of trained surveyors.

Participants were informed about the objective of the study and of their rights to data confidentiality before they filled the questionnaire. The study also implemented and fulfilled ethical research principles and obtained due permission from the Research Ethics Committee of the Faculty of Psychology at Universitas Airlangga.

Data analyses were performed via SPSS 26 for Windows. Regression analysis was used to test the hypotheses, and Bayesian ANOVA and bootstrapping ANOVA were employed to answer the explorative research question.

### 2.3 Instruments

This study utilised two instruments: a religious schema scale (RSS) and a tolerance scale. Streib et al.’s (2010) RSS was employed. It comprises three dimensions: the truth of text and teachings (five items; α = .897; sample item: ‘The texts and stories of my religion are absolutely true and must not be changed’); FTR choice (five items; α = .878; sample item: ‘It is important to understand others and attain a sympathetic understanding of their cultures and religions’) and xenosophia (five items; α = .805; sample item: ‘We must look beyond the denominational and religious differences to discover the ultimate reality’). Responses were rated on a scale ranging from 1 (highly disagree) to 7 (highly agree).

Hook et al.’s (2017) tolerance scale was adopted from a study that investigated the open-mindedness and tolerance displayed by residents of the United States towards non-Christian religions. Hook et al. (2017) implemented statements developed by Putnam and Campbell (2012), using four items pertaining to participant attitudes towards a specific group. These items were also utilised by the current study (e.g. ‘to what extent do you believe individuals from the following groups can go to heaven or attain salvation’), which added a further item, ‘these individuals can be good friends of mine’. The current study specifically targeted believers in indigenous faiths (α = .881) and atheists (α = .877). In sum, five items were utilised to query attitudes towards each target group. Responses were rated on a scale of 1–9 (1 denoted the most negative response to the item, and 9 represented the most positive). Hook et al.’s (2017) instrument (2017) was selected because it aligned Mummendey and Wenzel's (1999) definition of tolerance, and some items explicitly indicated an openness to outgroups. This scale also incorporated the traits of empathy and consideration of the sufferings of others, which denote key aspects of tolerance, according to Witenberg (2007; 2019).

## 3 Results

Data categorisation was accomplished based on a hypothetical norming by calculating the possible score range (range = from maximum to minimum scores), the hypothetical mean ($\text{μ }=\frac{maximum\;score\;+\;mininum\;score}{2}$) and the hypothetical standard deviation ($\text{σ}=\frac{range}{6}$) of the scale. Scores (x) were classified as very low when x ≤ μ – 1,5σ; as low when μ – 1,5σ < x ≤ μ – 0,5σ; as moderate when μ – 0,5σ < x ≤ μ + 0,5σ; as high when μ + 0,5 σ < X ≤ μ + 1,5σ and as very high when μ + 1,5σ < X.

Based on the formula above, the score categorisation for all dimensions of religious schema (i.e., TTT, FTR, XENOS) was illustrated: very low (x ≤ 2.5), low (2.5 < x ≤ 3.5), moderate (3.5 < x ≤ 4.5), high (4.5 < x ≤ 5.5) and very high (5.5 < x). In similar manner, the classification of the tolerance score was calculated as very low (x ≤ 3), low (3 < x ≤ 4.3), moderate (4.3 < x ≤ 5.7), high (5.7 < x ≤ 7) and very high (7 < x) (see Table 1).

**Table 1:** Data categorisation.

Variable	Category				
	Very low	Low	Moderate	High	Very High
The truth of texts and teachings	37	45	106	139	434
Fairness, tolerance and rational choice	20	7	19	103	612
Xenosophia	21	47	145	242	306
Tolerance towards believers of indigenous faiths	59	70	182	202	248
Tolerance towards atheists	253	117	173	145	73

Pearson's correlational analyses were run before undertaking the regression analysis (see Table 2). The results indicated that tolerance towards believers of indigenous faiths was associated with FTR and XENOS. However, tolerance towards atheists was only significantly linked with TTT. Therefore, multiple regression analysis was then performed to evaluate the contribution of these significant variables (see Table 3).

**Table 2:** Intercorrelations between variables.

	M	SD	1	2	3	4
Tolerance towards traditional beliefs (1)	6.08	1.94	—			
Tolerance towards atheism (2)	4.28	2.12	.258∗∗	—		
The truth of texts and teachings (3)	5.37	1.42	−.002	−.341∗∗	—	
Fairness, tolerance and rational choice (4)	5.99	1.08	.106∗∗	.053	.498∗∗	—
Xenosophia (5)	5.09	1.18	.089∗	.051	.374∗∗	.577∗∗∗

*Note.* ∗p < .05, ∗∗p < .01, ∗∗∗p < .001.

**Table 3:** Regression analysis of determinant variables for the prediction of tolerance.

Outcome	Predictor		
	TTT	FTR	XENOS
Tolerance towards indigenous beliefs			
B		.148	.069
SE B		.079	.072
B 95% CI		−.008,.304	−.073,.211
β		.082	.042
Tolerance towards atheism			
B	−.508∗∗		
SE B	.051		
B 95% CI	−.608, -.408		
β	−.341∗∗		

*Note.* ∗∗p < .01.

Assumption checking was performed separately for each regression model with tolerance for the two alienated groups (i.e., believers of indigenous faiths and atheists) as the outcome variables. The results indicated no multicollinearity with the variance inflation factor for each predictor ranging from 1 to 1.49, and the degree of tolerance varied between 0.66 and 1. Residuals were normally distributed as indicated by the P–P plots. No autocorrelation was found through Durbin–Watson values in both regression models ranging from 1.67 to 1.90. Cook's distance values evinced no substantial effects of any case or outlier on the regression models.

The result of the F-test on Model 1 was significant (i.e. tolerance for believers of indigenous faiths as the outcome variable); however, no single predictor (i.e. FTR and XENOS) significantly contributed to the regression model F (2, 758) = 4.78, p = .009, R^2^ = .01, effect size *f*^*2*^ = .01). Thus, H1 was not confirmed.

**Figure 1:**
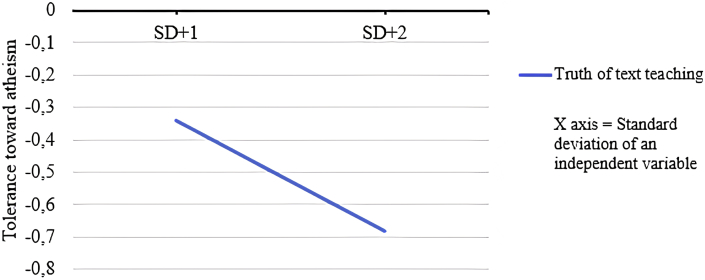
Standardised coefficient beta regression.

Model 2 (i.e. tolerance towards atheists as the outcome variable) revealed that TTT significantly explained 12% of the total variance of tolerance towards atheism F (1, 759) = 100.08, p = .000, R^2^ = .117, effect size *f*^*2*^ = .132). A high level of cognitive schema related to the truth of religious texts and teachings (B = −.508, 95% confidence intervals (CI) [−.608, −.408], SE = .05, t = −10.004, p = .000) would contribute to a low degree of tolerance for atheism. H2 was therefore partially confirmed because only one dimension of the religious schema (i.e. TTT) was a significant predictor (see Figure 1), while the other two (i.e. FTR and XENOS) were not.

**Figure 2:**
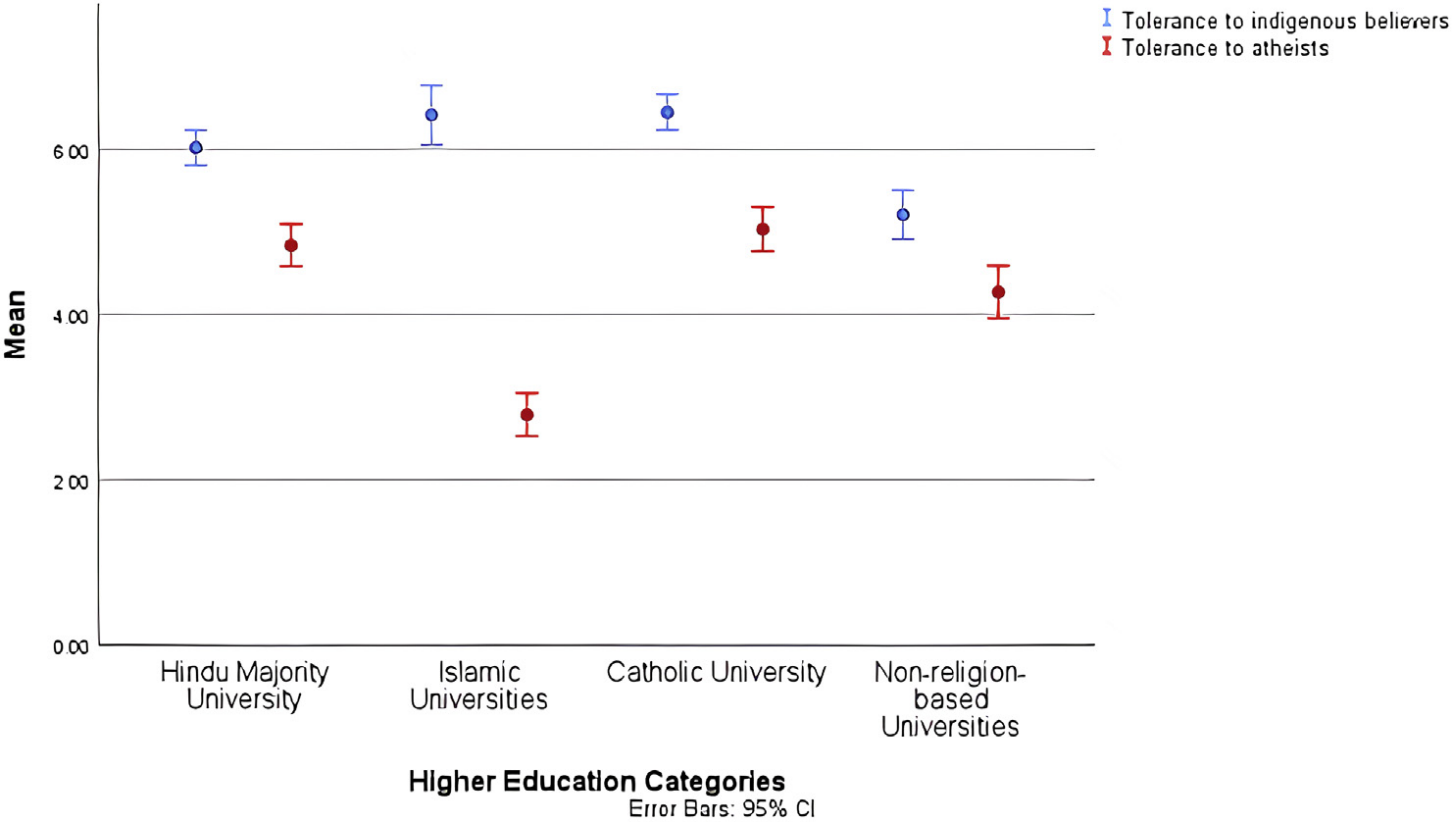
Comparison of tolerance towards alienated groups across university cohorts.

A Bayesian ANOVA was conducted as a test of the mean difference because of the unequal sample sizes of the university groups. The results indicated differences in tolerance levels registered towards believers of indigenous faiths among the four university groups: F (3, 757) = 14.053, p = .000, Bayes factor (JZS) = 59573.345, eta-squared = .052. The bootstrapping ANOVA (Table 4) and categorisation using the Ryan–Einot–Gabriel–Welsch range demonstrated that the non-religion-based universities evidenced a stark mean difference (lower) from the other university groups in terms of their tolerance towards believers in indigenous. The bootstrap 95% CIs also indicated areas in which the lower and the upper bound CIs of Islamic universities and non-religion-based universities did not overlap (see Figure 2).

**Table 4:** University-based differences in tolerance towards believers of indigenous faiths and atheists (the results of bootstrapping ANOVA).

Dimensions	Tolerance								
	Indigenous Beliefs				Atheism				
	Mean categorised based on homogenous subset∗		Bootstrap BCa 95%CI	SD	Mean based on homogenous subset∗			Bootstrap BCa 95%CI	SD
	1	2			1	2	3		
Public university with Hinduism as the major religion		6.019	5.829, 6.209	1.706			4.835	4.578, 5.081	2.054
Islamic universities		6.417	6.049, 6.779	2.482	2.788			2.528, 3.040	1.804
Catholic university		6.450	6.220, 6.666	1.535			5.031	4.738, 5.313	1.918
Non-religion-based universities	5.208		4.890, 5.533	1.762		4.270		3.947, 4.581	1.897

*Note.* ∗ classified through the Ryan–Einot–Gabriel–Welsch range.

The results of the Bayesian ANOVA also demonstrated significant differences among the four university groups in terms of their tolerance for atheists: F (3, 757) = 53.418, p = 0.000, Bayes factor (JZS) = 2.524E + 27 eta-squared = .174. The results of the bootstrapping ANOVA (Table 4) and categorisation using the Ryan–Einot–Gabriel–Welsch range evinced three category means for which the lower and upper bound CIs did not overlap with each other. The three categories of the levels of tolerance towards atheists are illustrated in Figure 2. Islamic universities demonstrated low levels of tolerance, non-religion-based universities displayed moderate levels of tolerance, and the university with Hinduism as its major religion and the Catholic university recorded upper-middle levels of tolerance.

## 4 Discussion

The study findings evidenced dissimilarities in the significance of each dimension of the religious schema as a predictor of tolerance towards alienated groups. None of the three religious dimensions could significantly forecast tolerance towards believers of indigenous faiths. However, the TTT dimension was a significant predictor of tolerance towards atheists. Streib and Klein's (2014) study elucidated that the significance and the prognostic power of each dimension of the religious schema depended on the target group in question (e.g. anti-Islamic vs. anti-Semitic prejudices).

The current study found a distinct association between TTT and tolerance towards atheists. People who believed strongly in the truth inscribed in their religious scriptures were likely to exhibit lower tolerance towards atheists. A number of other studies have also reported similar findings: the more literally people interpret their religious teachings, the higher their prejudice against outgroups and the lower their tendency to engage with other religions (Ardi and Budiarti, 2020; Hunsberger and Jackson, 2005; Küpper and Zick, 2011; Wrench et al., 2006). Ardi and Budiarti's (2020) study demonstrated a negative association between religious fundamentalism and contact with outgroups. Religious scriptures offer a frame of reference through which believers can interpret reality so that they can establish goals and build relationships with other groups (Hunsberger and Jackson, 2005). In the present instance, religious fundamentalism denotes the tendency to interpret one's religious teachings in a literal, rigid, and dogmatic fashion.

Interestingly, the FTR and XENOS dimensions of the religious schema did not significantly predict tolerance for either atheism or believers of indigenous faiths. This finding actually supported the assumption postulated by Jennings and Ralph-Morrow (2020) that tolerance is often selectively and strategically manifested in certain groups, and its expressions are not generally sincere. For instance, the right-wing group in Europe claims it is not intolerant towards any minorities, but particular clusters of people such as the liberals, LGBTQ communities and the followers of Islam are still stigmatised. The majority group often adopts a protective mechanism to defend itself from outgroups perceived as threats (Glaeser, 2005; Jennings and Ralph-Morrow, 2020).

Nevertheless, a few points about the nature of the three religious schema dimensions must be considered: 1) TTT is the most rigid schema governing individual opinions based on the absolute belief in the scriptures of a particular religion; 2) In contrast, FTR and XENOS concern personal perspectives regarding oneself and one's religion (including the ingroup) as well as an understanding of other groups as indicated by the FTR dimension items of the RSS. Further, the items of the XENOS dimension include individual attempts to engage in meta-reflection through which interaction with others allows people to reflect more intensively on their beliefs and on the actions of others. In other words, the three dimensions of the religious schema differ in their orientations vis-à-vis others. TTT is internally oriented towards personal faith, while the other two dimensions are externally directed towards interactions with other people. Jennings and Ralph-Morrow (2020) asserted that the differences in significance between the associations of the RSS dimensions with tolerance could be attributed to individual tendencies to choose certain ‘worthy’ outgroups for acceptance. All groups can claim that they are tolerant. In reality, however, they all exhibit preferences about which outgroups are or are not tolerable.

The inconsistencies in the significance of the associations between religious schema and tolerance towards alienated groups can be explained through an examination of how a schema is shaped. Schemata related to social interactions are formed through direct experiences or schema-related information exchanges (Nishida, 1999). A schema is stored in an individual's brain if that person engages with or receives information about alienated groups. This schema becomes more organised, abstract, compact, and readily available with increased contact and information reception (Nishida, 1999). This elucidation is indeed speculative, as the current study did not probe the extent of the respondents' interactions with the targeted minorities. Nevertheless, most participants could be assumed to live in homogeneous environments (i.e. religion-based universities wherein the majority practice only the mainstream religions). Logli (2015) also reported the homogeneity of demographic attributes in some Indonesian universities, pertaining particularly to religion, race, and socioeconomic strata. Hence, it is likely that the study participants only interacted with familiar groups.

Notably, atheism exists in Indonesia but is not readily detectable because it is not recognised by the state (Duile, 2018; Farhan and van Klinken, 2020; Schäfer, 2016). The absence of direct interactions allows only minimal or limited development of schema about atheism. The situation is certainly different from the relationships established between the state-recognised religions (i.e. Islam, Christianity, Catholicism, Hinduism, Buddhism and Confucianism). Most Indonesian people are likely to have had interactions with or are familiar with people practising these religions. The national recognition of religions also manifests in the observance of religious holidays (Ropi, 2017). It is assumed that cognitive schemas become readily accessible as an outgroup becomes more familiar for an individual; however, the cognitive schema is likely to be limited or totally unavailable when a group is severely alienated (Hogg and Vaughan, 2018; Murphy et al., 1995; Zajonc, 1968). Perhaps, this rationale explains why FTR and XENOS did not significantly predict tolerance towards atheism: the participants had not formed a clear cognitive schema about this alienated group.

Conversely, the indigenous beliefs are now officially recognised by the Indonesian government, but their religious manifestations could still be perceived as ambiguous by most citizens. In some contexts, indigenous religions are often called *Islam abangan* (i.e. a syncretism between Islam and the rituals of traditional religions such as Hinduism and Buddhism) (Hasbunallah, 2019; Qomar, 2015). Indigenous religious teachings are actually derived from the negotiations and interactions between the domains of discrete religions or groups (Leopold and Jensen, 2016; Munawar-Rachman, 2011). Additionally, the believers of indigenous faiths cannot be reduced to a singular entity or one group with different denominations. Instead, they represent multiple groups with diverse faith systems (Ali-Fauzi et al., 2011; DKT, 2017). Almost every region has its version of a traditional belief system, for example, Sunda Wiwitan in Sunda, Kejawen in Java, Parmalim in North Sumatera and so on (DKT, 2017; Mu'ti & Burhani, 2019). The majority community still perceives these faiths in an indeterminate manner as variations of the mainstream religions, syncretism or completely different religions (Hasbunallah, 2019; Leopold and Jensen, 2016; Qomar, 2015). Perhaps, this ambiguity of perspective could explain why none of the religious schema dimensions was found to significantly predict tolerance towards this group.

The assumption about the general population's minimal familiarity with the targeted alienated groups was implicitly supported by the results of the mean difference testing performed for this study. The analysis of the tolerance displayed towards the atheism-related data yielded three distinct clusters. Participants from the university with Hinduism as the major religion and from the Catholic university presented a high level of tolerance, followed by students of non-religion-based universities who demonstrated a moderate level of tolerance. However, participants belonging to Islamic universities exhibited lower levels of tolerance in comparison to other university groups. A survey conducted by PPM UIN Jakarta (2021) also documented a similar finding that Muslim university students perceived higher levels of threat and reported lower levels of intergroup social interactions in comparison to students of other religions.

The high levels of tolerance reported by students in Bali could also be attributed to their social demographical characteristics. The majority of people in Bali universities adhere to Hinduism; further, Bali is a cosmopolitan region where students are most likely to have interacted with diverse groups representing cultures worldwide (Arumsari et al., 2020). This multiculturalism is driven by Bali's tourism industry and allows increased opportunities for students to interact with people from varied backgrounds. In turn, such exchanges allow transformations in discourses and self-identities (Connor and Vickers, 2003). Moreover, Hinduism predominates in Bali but is a minority religion on the national scale. Inter-minority solidarity towards stigmatised alienated groups may thus make Hindus more tolerant and accepting of other minority groups (Ball and Branscombe, 2019).

Inter-minority solidarity could also explain the high level of tolerance reported by Catholic university students towards atheism and believers of indigenous religions. Categorising oneself as a fellow minority could result in a feeling of moral obligation and create a sense of unity with other minority groups experiencing the same fate (Ball and Branscombe, 2019; Glasford and Calcagno, 2012).

In contrast, the low tolerance for atheism expressed by students of Islamic universities could be attributed to homogeneity and to their educational climate. The two Islamic universities whose students were engaged in the current study were located outside Indonesia's metropolitan and cosmopolitan areas. The students attending these institutions are less likely to have contact with dissimilar groups. This outcome is congruent with PPM UIN Jakarta's (2021) report that the social climate of the campus and tolerance levels of the faculty members positively influence the tolerance levels of students. PPM UIN Jakarta (2021) also stated that Muslim students registered low levels of intergroup social interactions.

In general, all university groups evinced higher levels of tolerance towards indigenous faiths than towards atheism. Interestingly, participants attending Islamic universities exhibited a higher level of tolerance towards believers in indigenous faiths than towards atheists. It could thus be assumed that traditional beliefs are perceived as tolerable aspects because the country has officially recognised them. Moreover, some indigenous faiths also practice some Islamic values. One indigenous group even proclaims itself *Islam Kejawen* (i.e. Islam syncretised with Javanese traditional spiritual practices) (Hasbunallah, 2019; Qomar, 2015).

Students from universities unaffiliated to any particular religion displayed moderate levels of tolerance towards the two alienated groups. Their level of tolerance was lower than the acceptance demonstrated by the Bali and Catholic universities. This finding was unanticipated, as these secular universities are located in a major city in Indonesia. The heterogeneity of these institutions presumably caused students to become ambiguous in their attitudes. Perhaps, they sensed a tension between accepting atheism and denying a group unrecognised by the country's ideology. In terms of tolerance for the believers in indigenous faiths, it has been noted above that they cannot be viewed as a single entity because they represent diverse and plural groups (Ali-Fauzi et al., 2011). Moreover, the formation of these beliefs is often unnoticed as an aspect of sociocultural interactions (Leopold and Jensen, 2016). The indigenous religions can be perceived as completely different traditional beliefs, an assimilative syncretism, or a part of the mainstream religions (Hasbunallah, 2019; Qomar, 2015). On the one hand, the heterogeneous interactions within secular universities enable individuals from all backgrounds to meet each other. On the other hand, the unaffiliated university bodies can also evince the plurality of the indigenous faiths to their students, who can then perceive them as diverse entities that cannot be generalised. Thus, one indigenous belief could be perceived as sensible and acceptable, while others are not.

## 5 Conclusion

Disparities were noted in the significance of the dimensions of the religious schema vis-à-vis tolerance for alienated groups. The social and educational climate of a university also determined the level of tolerance evinced by its student body towards such groups.

The insights attained from these outcomes allow the assertion that future research projects on the associations between religious schema and tolerance towards certain groups should also consider the familiarity participants feel apropos the target groups because tolerance could be selectively and strategically developed towards specific minorities. Additionally, future studies should also consider the degree of inter-minority solidarity when involving participants from minority groups. It is pertinent to examine whether the perception of a shared destiny can influence a person's acceptance of other alienated groups.

Further, prospective research endeavours can investigate the mediating and/or moderating role of religious schema in the relationships between types of universities and tolerance. Such investigations would interest scholars given the present study's findings of links between types of universities and tolerance levels as well as between religious schema and tolerance levels. Finally, this study attended only to the assumption that the homogeneous and/or heterogeneous interactions that occur within a particular type of university would partly determine the level of tolerance of its students. Admittedly, the individual predispositions to tolerance towards outgroups before students were admitted to the universities were not explored. Follow-up studies can investigate the associations between internal predispositions related to outgroup tolerance and choice of university.

The study findings can aid in policy-making in universities and governmental bodies (both on regional and national levels) to improve tolerance towards alienated groups. This study also recommends that mass media should increase coverage and should proliferate public dialogues about the values of diversity so that the understanding, acceptance, and tolerance towards all groups, including minorities and the most alienated ones, can be improved.

## Declarations

### Author contribution statement

Rahkman Ardi: Conceived and designed the experiments; Analyzed and interpreted the data; Wrote the paper.

David Hizkia Tobing, Gita Nuraini Agustina, Ahmad Fauzan Iswahyudi, Diah Budiarti: Performed the experiments; Wrote the paper.

### Funding statement

This work was supported by the 10.13039/501100010447Ministry of Research, Technology and Higher Education of the Republic of Indonesia (Kemenristekdikti) [764/UN3.14/LT/2019].

### Data availability statement

Data included in article/supplementary material/referenced in article.

### Declaration of interests statement

The authors declare no conflict of interest.

### Additional information

No additional information is available for this paper.

## Appendix A Supplementary data

SURVEI NILAI RELIJIUSITAS DATASET SURVEI NILAI RELIJIUSITAS DATASET
